# Deferoxamine regulates neuroinflammation and iron homeostasis in a mouse model of postoperative cognitive dysfunction

**DOI:** 10.1186/s12974-016-0740-2

**Published:** 2016-10-12

**Authors:** Yuping Li, Ke Pan, Lin Chen, Jiao-lin Ning, Xiaojun Li, Ting Yang, Niccolò Terrando, Jianteng Gu, Guocai Tao

**Affiliations:** 1 Department of Anesthesiology, Southwest Hospital, Third Military Medical University, 30 Gaotanyan Road, Chongqing, 400038 China; 2 Department of Medicine, Division of Nephrology, Duke University Medical Center, Durham, 27710 NC USA; 3 Department of Anesthesiology, Basic Science Division, Duke University Medical Center, Durham, 27710 NC USA

**Keywords:** Cytokines, Hippocampus, Iron, Microglia, Surgery

## Abstract

**Background:**

Postoperative cognitive dysfunction (POCD) is a common complication after surgery, especially amongst elderly patients. Neuroinflammation and iron homeostasis are key hallmarks of several neurological disorders. In this study, we investigated the role of deferoxamine (DFO), a clinically used iron chelator, in a mouse model of surgery-induced cognitive dysfunction and assessed its neuroprotective effects on neuroinflammation, oxidative stress, and memory function.

**Methods:**

A model of laparotomy under general anesthesia and analgesia was used to study POCD. Twelve to 14 months C57BL/6J male mice were treated with DFO, and changes in iron signaling, microglia activity, oxidative stress, inflammatory cytokines, and neurotrophic factors were assessed in the hippocampus on postoperative days 3, 7, and 14. Memory function was evaluated using fear conditioning and Morris water maze tests. BV2 microglia cells were used to test the anti-inflammatory and neuroprotective effects of DFO.

**Results:**

Peripheral surgical trauma triggered changes in hippocampal iron homeostasis including ferric iron deposition, increase in hepcidin and divalent metal transporter-1, reduction in ferroportin and ferritin, and oxidative stress. Microglia activation, inflammatory cytokines, brain-derived neurotropic factor impairments, and cognitive dysfunction were found up to day 14 after surgery. Treatment with DFO significantly reduced neuroinflammation and improved cognitive decline by modulating p38 MAPK signaling, reactive oxygen species, and pro-inflammatory cytokines release.

**Conclusions:**

Iron imbalance represents a novel mechanism underlying surgery-induced neuroinflammation and cognitive decline. DFO treatment regulates neuroinflammation and microglia activity after surgery.

**Electronic supplementary material:**

The online version of this article (doi:10.1186/s12974-016-0740-2) contains supplementary material, which is available to authorized users.

## Background

Neurological complications after major surgery are common, especially in a rapidly growing aging population [1, 2]. The most common long-term postoperative complication within this large patient group is a reduction in thinking and memory processes termed postoperative cognitive dysfunction (POCD, recently reviewed in [3]). POCD affects up to 14–24 % of patients following non-cardiac surgery and increases the risk for further complications, including mortality, and prolonged hospitalization quickly becoming a significant burden to the health care system [3, 4]. Currently, there is no evidence-based treatment for POCD.

Inflammation is gaining considerable interest as a critical driver of cognitive deficits, including neurodegenerative conditions like Alzheimer’s disease (AD) [5, 6]. Neuroinflammation has been related to models of surgery-induced cognitive dysfunction, in particular, the release of pro-inflammatory cytokines as tumor necrosis factor-alpha (TNF-α) and interleukin-1 beta (IL-1β) and the activation of nuclear factor-kB (NF-kB) signaling in macrophages and microglia have been highlighted as critical factors in the development of cognitive deficits [7–9]. Changes in pro-inflammatory cytokines and neurodegenerative markers in the cerebrospinal fluid of postsurgical patients have been similarly detected, suggesting a role for neuroinflammation in the pathophysiology of POCD [10, 11]. Furthermore, animal models have reported a correlation between pro-inflammatory cytokines and synaptic plasticity, which is the substrate for memory formation in the hippocampus [12, 13].

Iron homeostasis is fundamental in maintaining central nervous system (CNS) function and is a necessary factor in the regulation of oxygen transport, neurotransmission, myelination, and neuronal metabolism [14]. However, iron imbalance and aberrant accumulation in the CNS is also a hallmark of neuroinflammation and is implicated in several neurodegenerative disorders, including AD [15, 16]. Mounting evidence supports the idea that iron progressively accumulates in the brain with age, leading to oxidative stress, cell death, and neurotoxicity [14]. As a consequence of this microenvironment, microglia become *primed*, thus sensitized to a subsequent challenge (i.e., infection or trauma) and may contribute to chronic non-resolved neuroinflammation [17, 18].

Although aging is one of the key risk factor for POCD, strategies aimed at reducing postoperative neuroinflammation have been limited [19, 20]. Hence, this study aimed at providing new evidence for iron dysregulation as a potential target for postoperative neuroinflammation in a clinically relevant POCD model. Herein, we explored the effects of deferoxamine (DFO), a potent iron chelator agent, in preventing microglia activation and hippocampal-dependent memory deficit after laparotomy in mice. These findings provide evidence for iron accumulation and activation of p38-mitogen-activated protein kinase (MAPK) signaling in microglia after surgical trauma as a novel target to treat POCD.

## Methods

### Animals

C57BL/6J male mice (12~14 months) were obtained from the Changzhou SPF Animal Technology Co. Ltd (Changzhou, China). Mice were housed in a controlled environment (20 ± 2 °C and 50 ± 10 % humidity, 12:12 light/dark cycle) with ad libitum access to standard chow and water. The procedures were approved by the Committee of Ethics on Animal Experiments at Southwest committee of laboratory animal committee of PLA, Chongqing, China (protocol number: SYXK20120031), and followed the guidelines for the care and use of Laboratory Animals.

### Experimental protocol and surgery model

**Fig. 1:**
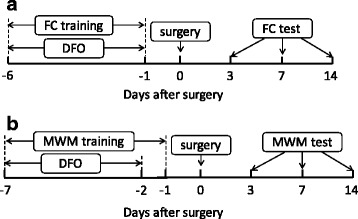
Experimental protocol. **a** Protocol of contextual fear conditioning. **b** Protocol of Morris water maze (a separate cohort of mice). *DFO* deferoxamine, *FC* fear conditioning, *MWM* Morris water maze

Mice were treated daily for 6 days with 100 mg/kg DFO (Sigma-Aldrich, Inc., St. Louis, MO, USA) intraperiotoneally (i.p.) [21]. During the DFO administration days, the training trials or behavior tests were performed as described below. Abdominal exploratory surgery [22] was then performed 24 h after the behavior trainings. The experimental protocols are indicated as in Fig. 1.

On the day of surgery, mice were anesthetized with 4 % chloral hydrate (10 ml/kg, i.p., Shanghai Xingya Medical Company) plus 0.1 % lidocaine (Shanghai Zhaohiu Pharm Co. Ltd, China). Briefly, the gastrointestinal tract was exteriorized, and abdominal organs (liver, spleen, kidneys, and bowel) were explored gently with cotton but for 30 min. The abdomen was then closed by 8/0 Prolene sutures (CE1023 Jinhuan Co. Ltd, Shanghai, China). 0.1 % lidocaine was also used for postoperative analgesia. Body temperature was maintained at 37 ± 0.5 °C using a homeothermic blanket for rodents (Stoelting, USA). Sham mice were exposed to anesthesia and received a midline abdominal incision (~3 cm) without manipulation of other organs. Mice without surgery served as naïve controls.

#### Behavioral tests

##### Fear conditioning

Contextual fear conditioning (FC) test was performed in a dedicated chamber (Biowill Co. Ltd, Shanghai, China) as previously described [23]. Six hours after the daily DFO administration, mice were placed in FC chamber to adapt to the context for 2 min. The conditional stimuli cycle was then applied as 15 s tone (80 dB)—30 s delay—5 s electrical foot shock (0.3 mA). The conditional training was repeated for six consecutive days. Surgeries were then performed 24 h after the last day of training. On postoperative days 3, 7, or 14, mice were placed back in the original conditioning chamber without tone or shock stimuli. The recall of contextual fear memory was assessed by freezing behavior (Freeze Frame Actimetrics software). The experimental protocol was indicated as in Fig. 1a.

##### Morris water maze

The Morris water maze (MWM) training was continued in a separate cohort of mice for seven consecutive days before surgery. DFO was administered 6 h before MWM daily, from the first to sixth training days. The water maze tank was 120 cm in diameter, 30 cm in depth, and filled with water at 22 °C. A submerged platform (10 cm in diameter) was located at a fixed location which was in the target quadrant. The mouse was released into the water facing the wall of the pool from one quadrant and allowed 60 s to locate the hidden platform [24]. If a mouse failed to find the platform within 60 s, it was guided to the platform and placed on the platform for 15 s. The mouse was then removed to the cage and allowed to dry in a warm environment. Four trials were performed on each mouse with starting location from different quadrant. Twenty-four hours after the MWM training, abdominal exploration or sham surgery were performed. The acquisition tests and probe trial were performed on postoperative days 3, 7, and 14. In the end of MWM tests on postoperative day 14, the mice were terminated for organ harvest. The latency to reach the platform and swimming speed, as well as the proportion of time spent in target quadrant and platform crossing times during the probe trials were recorded and analyzed by video tracking system (Xinruan Information Technology Co. Ltd, Shanghai, China). The experimental protocol was performed as described in Fig. 1b.

### Immunohistochemistry

On postoperative days 3, 7, and 14, mice were anesthetized with 4 % chloral hydrate and 0.1 % lidocaine and transcardially perfused with 0.9 % ice-cold saline followed by 2.5 % paraformaldehyde (PFA, Beyotime Institute of Biotechnology, Shanghai, China). Brains were harvested and postfixed in 2.5 % PFA. The samples were then cryoprotected in 30 % sucrose solution and embedded in optimal cutting temperature compound (OCT, Xingzhi Biological technology co., LTD, Guangzhou, China). Coronal sections (25 μm) were obtained with a cryostat (Leica, Germany). Sections were blocked in 10 % normal goat serum (Beyotime Institute of Biotechnology) for 30 min at room temperature and incubated with a rabbit Iba1 antibody (1:500, Wako, Japan) at 37 °C for 1.5 h and then at 4 °C overnight. After PBS wash, sections were incubated in goat anti-rabbit antibody (1:300, Beyotime Institute of Biotechnology, China) for 1 h at 37 °C and then in horseradish peroxidase streptavidin (1:200) and visualized with DAB kit (both from Beyotime Institute of Biotechnology). Images were obtained with a microscope (Leica, Wetzlar, Germany). Iba1-positive cells were analyzed using Image-Pro-Plus® 6.0 Software; the cell body to cell size ratio was used to assess microglial activation [25].

### Oxidative stress assays

Reactive oxygen species in hippocampus were detected using a ROS Assay Kit (Nanjing Jiancheng Bioengineering Institute, China) following the manufacturer’s instructions. In brief, the hippocampus was homogenized with 100 mmol/L PBS and centrifuged at 1000*g* for 10 min at 4 °C. The supernatant was collected, and protein concentration was determined using Coomassie brilliant blue method of protein assay kit (Nanjing Jiancheng Bioengineering Institute, China). One hundred ninety microliter supernatant was added into 10 μl 2,7-dichlorofuorescin diacetate (1 mmol/L, DCFH-DA), and samples were incubated at 37 °C for 30 min. The fluorescence (λexc = 502 nm, λem = 530 nm) was monitored after the stabilization of the signal, and the results were expressed as fluorescence intensity/100 mg protein.

The malodialdehyde (MDA) level in hippocampus was determined using a MDA Assay Kit (Nanjing Jiancheng Bioengineering Institute, China). After protein quantification, 100 μl tissue homogenate, 100 μl standard solution (10 nmol/ml), 100 μl absolute ethyl alcohol, and 100 μl glacial acetic acid (50 %) were added into four reaction tubes, and then added the corresponding reagents following the manufacturer’s instruction. The reaction mixture was then heated at 95 °C for 40 min, centrifuged at 3500*g* for 10 min at room temperature. The absorbance was determined by Multi-function meter at 532 nm. The results were expressed as nanomole per milligram protein.

### BV2 microglial cells

The murine microglial cell line BV2 was kindly provided from Dr. He (Department of Neurobiology, College of Basic Medical Sciences, Third Military Medical University). DMEM (high glucose, Gibco, Grand Island, NY) supplemented with 5 % fetal bovine serum (FBS, Gibco), 100 U/mL penicillin, and 100 μg/mL streptomycin, in a humidified incubator at 37 °C supplied with 95 % air and 5 % CO_2_ [26]. Cells were treated for 16 h with (1) lipopolysaccharide (LPS, 20 μg/ml, Sigma), (2) DFO (5 mM), (3) DFO + LPS (with DFO pretreated for 6 h), and (4) DMSO (14 μM). DFO and LPS were dissolved in DMSO with final DMSO concentration at 14 μM. The concentration of LPS was chosen based on previous study [27]. BV2 cells cultured in 12-well plates (5 × 10^4^ cells/well) were used for western blot, and cells cultured in 96-well plates (4 × 10^4^ cells/well) were used for analysis of iron release or enzyme-linked immunosorbent assay (ELISA).

### Western blot

Hippocampus and BV2 cells were homogenized in lysis buffer (P0013, Beyotime Institute of Biotechnology, Shanghai, China) and centrifuged at 10,000*g* for 30 min at 4 °C. Protein concentration was determined using BCA protein assay kit (P0012, Beyotime Institute of Biotechnology). The samples were separated by 10 or 12 % SDS-polyacrylamide gel and transferred to polyvinylidene fluoride membranes. After 2 h blocking with 5 % skim milk at 37 °C, the membranes were incubated with primary antibodies (Table 1) overnight at 4 °C and then with secondary antibody (goat anti-rabbit or goat anti-rat antibody, 1:1000, Beyotime Institute of Biotechnology, Shanghai, China) for 2 h at 37 °C. The immunoreaction was visualized with enhanced chemiluminescence (ECL) detection reagents (Thermo Scientific, Rockford, IL, USA) and analyzed by Image Lab™ software (Bio-Rad Laboratories, Inc. Hercules, CA).

**Table 1:** Primary antibodies for western blot

Primary antibody	Concentration	Provider
Rabbit anti-ferroportin	1:1000	Abcam, Inc. Cambridge, UK
Rabbit anti-DMTI	1:500	Alpha Diagnostic Intl Inc
Rabbit anti-hepcidin	1:1000	Abcam, Inc. Cambridge, UK
Rat anti-CD68	1:500	Abcam, Inc. Cambridge, UK
Rabbit anti-BDNF	1:1000	Abcam, Inc. Cambridge, UK
Rabbit anti-ferritin	1:2000	Abcam, Inc. Cambridge, UK
Rabbit anti-p38	1:1000	Abcam, Inc. Cambridge, UK
Rabbit anti-gp91phox	1:1000	Abcam, Inc. Cambridge, UK

### Calcein-AM assay

The labile iron pool (LIP) was determined by a fluorescence technique with the Fe sensor calcein as previously described [28] with minor modifications. After washing with PBS, cells were treated with Chelex-100 (Bio-Rad Laboratories) and incubated with 100 μl Calcein-AM solution (final concentration at 30 μM) for 20 min at 37 °C. The excess Calcein-AM was washed off with PBS. The fluorescence (λexc = 450 nm, λem = 515 nm) was monitored after the stabilization of the signal, and results were expressed as fold change.

### Iron release assay

Iron concentration was measured by Iron Assay Kit (BioAssay Systems, USA). The procedure was performed as described [26] with minor modifications. The cells were crushed with soniprep (Ultrasonic cell crusher, SONICS, USA) after washing with PBS. Samples were incubated for 40 min at room temperature, and optical density was read at 590 nm. The results were expressed as fold change.

### Enzyme-linked immunosorbent assay

The concentrations of IL-1β, IL-6, and TNF-α in hippocampus and medium were examined by ELISA assay kits following manufacturer’s instructions (R&D Systems, USA). The hippocampus were homogenized in RIPA lysis buffer (Beyotime Institute of Biotechnology, China) and centrifuged at 12,000*g* for 15 min to obtain supernatant. Protein quantification was assessed using BCA kit following the instruction (Beyotime Institute of Biotechnology). BV2 cells were seeded in 96-well plates at 4 × 10^4^ cells/well for 24 h before the experiments. After treatment, the medium was collected after centrifugation at 1000*g* for 20 min and 100 μl of supernatant was used for detection. The absorbance was read using a spectrophotometer at a wavelength of 450 nm. The concentrations were calculated according to the standard curve and presented as picogram per milliliter.

### Statistical analysis

The data were expressed as mean ± standard deviation (SD) using GraphPad InStat software program. Two-group comparisons were evaluated by Student’s *t* test, and multiple comparisons were evaluated by one-way ANOVA followed by Bonferroni post hoc test. Separate two-way repeated-measures ANOVA was used to evaluate the effect of dose and time on each dependent variable in the fear conditioning and the MWM. *P* < 0.05 was considered statistically significant.

## Results

### Surgery affects iron homeostasis in the hippocampus

**Fig. 2:**
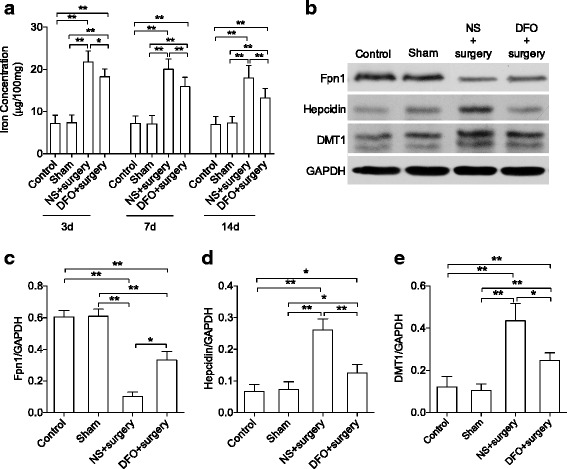
DFO attenuated surgery-induced iron increase in the hippocampus. **a** Hippocampal iron content on postoperative days 3, 7, and 14. **b**–**e** Western blot images and quantification of Fpn1, hepcidin, and DMT1 at day 14. *n* = 8/group for iron content, *n* = 6/group for iron marker, *P < 0.05, **P < 0.01; data are expressed as mean ± SD. *DFO* deferoxamine, *NS* normal saline, *Fpn1* ferroportin, *DMT1* divalent mental transporter1

Using an abdominal laparoscopy surgery model, we evaluated the impact of surgical trauma on iron homeostasis in the hippocampus. Surgery induced iron accumulation in the hippocampus compared to control and sham groups up to 14 days (*P* < 0.01), which was attenuated by DFO pretreatment (*P* < 0.05 and 0.01, respectively, Fig. 2a). To verify these changes, we measured protein expression of key components in iron homeostasis in the hippocampus including ferroportin (Fpn-1), hepcidin, and divalent metal trasporter-1 (DMT1). Compared to control and sham operation, surgery caused a significant reduction in Fpn-1, increasing both hepcidin and DMT1 in the hippocampus up to postoperative day 14 (*P* < 0.01). Notably, pretreatment with DFO significantly improved the surgery-induced changes in iron regulation (*P* < 0.05 and 0.01, respectively, Fig. 2b–e).

### DFO treatment prevents neuroinflammation and oxidative stress in the hippocampus

**Fig. 3:**
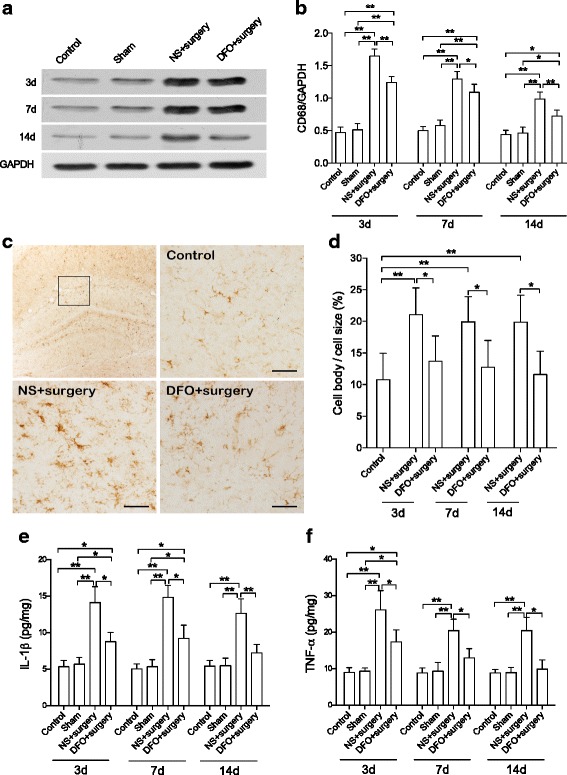
DFO reduced neuroinflammation in hippocampus. **a** Representative bands of western blot for CD68 in hippocampus. **b** Quantification of CD68 protein level in hippocampus. **c** Representative pictures of Iba1 staining in hippocampus. **d** The cell body/cell size ratio of Iba1-labeled microglia. **e**, **f** Hippocampal IL-1β and TNF-α. Surgery significantly increased the upregulation of microglia activation, as well as CD68, IL-1β, and TNF-α level in hippocampus. These effects were attenuated by DFO pretreatment. *n* = 4/group, *P < 0.05, **P < 0.01. Data are expressed as mean ± SD. *Scale bar* 50 μm. *DFO* deferoxamine, *NS* normal saline

Abdominal surgery led to significant increase of CD68 expression in hippocampus up to postoperative day 14 (*P* < 0.01, Fig. 3a, b). In addition, from postoperative days 7 to 14, surgery significantly increased Iba1-indicated microglial activity (*P* < 0.01, Fig. 3c, d) as well as the hippocampal TNF-α and IL-1β levels (*P* < 0.05 and 0.01, respectively, Fig. 3e, f). These changes were attenuated by DFO pretreatment (*P* < 0.05 and 0.01, respectively, Fig. 3). In addition, there is also significant correlation between hippocampal iron content and microglial activity (*r* = 0.6811, *P* = 0.0147) or CD68 level (*r* = 0.9561, *P* < 0.001, Additional file 1: Figure S1). The suggested neuroinflammation is strongly associated with iron content in the brain.

**Fig. 4:**
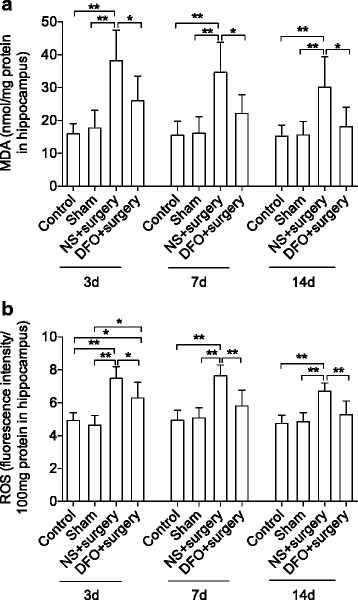
Effects of DFO on oxidative stress markers in the hippocampus. Surgery significantly elevated hippocampal levels of MDA (**a**) and ROS (**b**) on postoperative days 3 and 7. This effect was reduced by DFO pretreatment. *n* = 4/group, *P < 0.05, **P < 0.01. Data are expressed as mean ± SD. *ROS* reactive oxygen species, *MDA* malondialdehyde, *DFO* deferoxamine, *NS* normal saline

Aside neuroinflammation, the oxidative stress markers, MDA, and reactive oxygen species (ROS) in hippocampus were also significantly elevated from postoperative day 3 up to day 14 compared to naïve or sham-operated mice (Fig. 4a, b, *P* < 0.01), which were also significantly attenuated by DFO pretreatment (*P* < 0.05 and 0.01, respectively, Fig. 4a, b).

### DFO prevented surgery-induced BDNF dysfunction and memory impairments

**Fig. 5:**
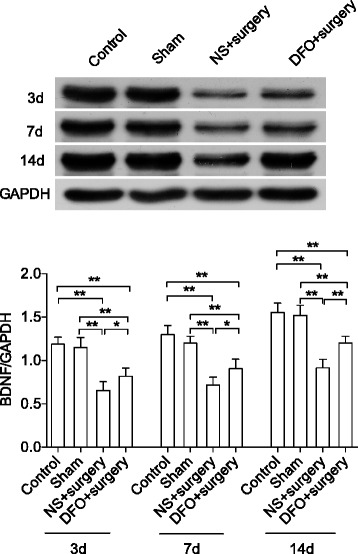
DFO improved BDNF expression after surgery. Image showed the representative western blot bands for BDNF. Surgery caused a reduction in BDNF protein, which was restored by DFO, as quantified using relative density. *n* = 4/group, *P < 0.05, **P < 0.01. Data are expressed as mean ± SD. *DFO* deferoxamine, *BDNF* brain-derived neurotropic factor, *NS* normal saline

**Fig. 6:**
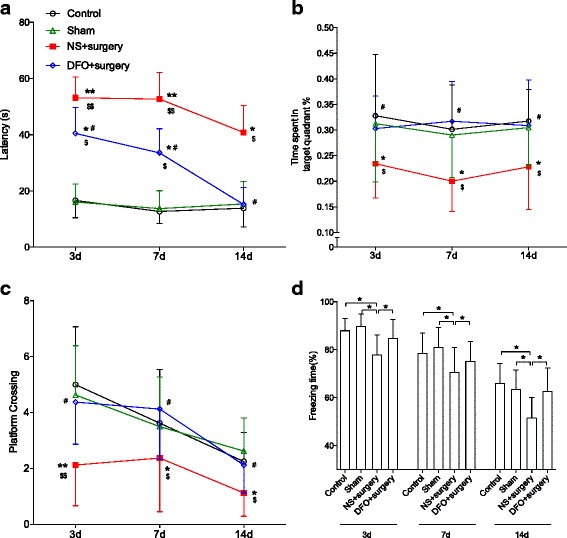
Surgery-induced hippocampal-dependent memory impairment is mitigated by DFO. Mice after surgery showed significant longer latency in MWM acquisition trials compared to naïve and sham-operated controls (**a**). Moreover, in probe trials, mice with surgery spent less time in target quadrant (**b**) and had fewer crossings over platform location (**c**) compared to controls. Contextual fear conditioning memories was impaired in mice after surgery compared to naïve and sham-operated controls (**d**). DFO pretreatment significantly improved the performance of mice in MWM and FC tests after surgery. *n* = 8/group, *P < 0.05, **P < 0.01 vs control group, ^#^ *P* < 0.05 vs NS + surgery group, ^$^ *P* < 0.05, ^$$^ *P* < 0.01 vs sham group. Data are expressed as mean ± SD. *DFO* deferoxamine, *NS* normal saline

Levels of brain-derived neurotropic factor (BDNF), a key trophic factor in the CNS, were significantly reduced after surgery from day 3 up to day 14 compared to control and sham-operated group (*P* < 0.01). This effect was significantly alleviated by DFO treatment (*P* < 0.05 and 0.01, respectively, Fig. 5). Next, we used FC and MWM tests to evaluate the cognitive function in this POCD model [23, 24]. During training, we found no difference between groups regarding the average latency per day; however, the calculated area under the curve was significantly reduced in the DFO-treated group compared to other groups (*P* < 0.05 and 0.01, respectively, Additional file 2: Figure S2) suggesting DFO may facilitate learning. During the testing trials, both control and sham groups maintained similar latencies as in the last training session whereas the surgery group spent more time locating the hidden platform up to postoperative day 14 compared to control and sham-operated mice (*P* < 0.05 and 0.01, respectively, Fig. 6a). DFO treatment significantly improved the latency compared to non-treated mice during testing trials at all time points (*P* < 0.05), suggesting that DFO attenuated surgery-induced memory impairment. Moreover, in the probe trial, mice from surgery group spent significantly less time in the platform target quadrant and less times crossing over the platform area (*P* < 0.05 and 0.01, respectively). DFO pretreatment significantly improved the performance during probe trial after surgery (Fig. 6b, c). No differences of swimming speed were observed between groups at all the time points (data not shown).

In FC, freezing time was significantly reduced after surgery at all time points (*P* < 0.05 vs control and sham groups, respectively). Treatment with DFO restored memory function with no difference compared to naïve or sham-operated mice up to postoperative day 14 (Fig. 6d).

### Anti-inflammatory effects of DFO in LPS-exposed microglia cells

**Fig. 7:**
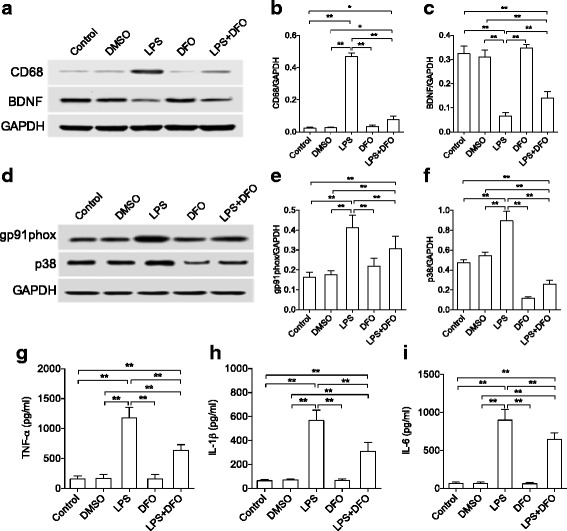
Anti-inflammatory effects of DFO on microglia cells in vitro. **a** Image of western blot protein bands for CD68 and BDNF in microglia cells. **b**, **c** Quantification of CD68 and BDNF protein levels. **d** Image of western blot protein bands for gp91phox and p38 in microglia cells. **e**, **f** Quantification of gp91phox and p38 protein levels. **g**-**i** Pro-inflammatory cytokines in microglia cells culture medium. DFO exerted anti-inflammatory effects on BV2 cells. *n* = 6/group except for p38MAPK (*n* = 4/group), *P < 0.05, **P < 0.01. Data are expressed as mean ± SD. *DFO* deferoxamine, *LPS* lipopolysaccharide, *BDNF* brain-derived neurotropic factor

We used BV2 cell line to further investigate the effects of DFO on microglia. DFO pretreatment significantly decreased LPS-induced CD68 expression (*P* < 0.01, Fig. 7a, b) and improved BDNF level following LPS stimulation (*P* < 0.01, Fig. 7a, c). In addition, LPS-induced gp91phox and p38-MAPK were significantly reduced by DFO pretreatment (*P* < 0.01, Fig. 7d, f). LPS also increased levels of IL-6, TNF-α, and IL-1β in cell culture media compared to naïve or DMSO alone group (*P* < 0.01). This effect was significantly reduced by DFO pretreatment (*P* < 0.01, Fig. 7g–i).

**Fig. 8:**
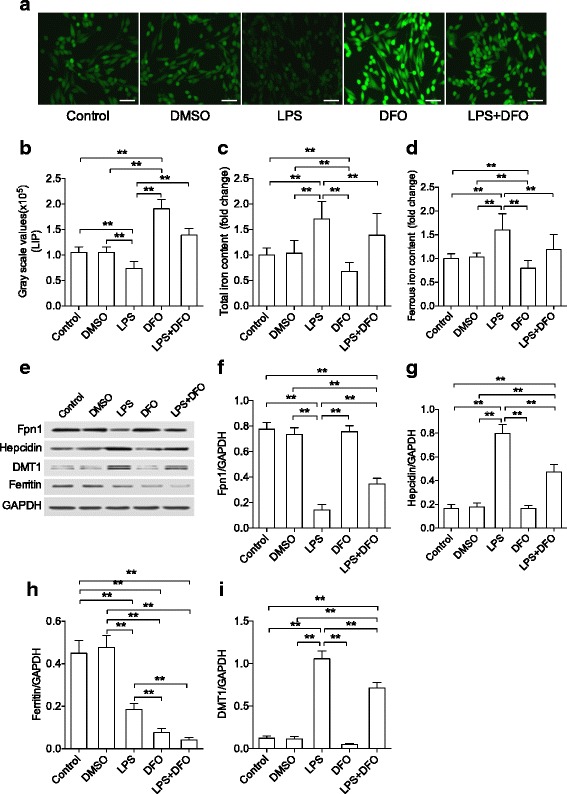
DFO regulates iron content and transport proteins in LPS-activated microglial cells. **a**, **b** Fluorescence staining of metal-sensitive probe calcein showed labile iron in microglia cells. DFO increased labile iron content in both resting and activated microglia cells. DFO also inhibited LPS-induced increase in total iron (**c**) and ferrous ion (**d**) content in microglia cells. Representative images of western blot protein bands of target proteins (**e**). DFO significantly attenuated LPS-induced changes in protein levels for Fpn1 (**f**), hepcidin (**g**), and DMT1 (**i**). Ferritin levels (**h**) were decreased in microglia cells after LPS or DFO treatments. *n* = 4/group for LIP, *n* = 6/group for total iron and iron marker, *n* = 8/group for ferrous ion, *P < 0.05, **P < 0.01. Data are expressed as mean ± SD. *Scale bar* 50 μm. *DFO* deferoxamine, *LPS* lipopolysaccharide, *Fpn1* ferroportin, *DMT1* divalent mental transporter1, *LIP* labile iron pool

Looking at iron signaling in BV2 cells, LPS stimulation significantly increased labile iron, total iron, and ferrous iron in microglial cells compared to naïve and DMSO control groups (*P* < 0.01). These effects were abolished by DFO pretreatment (*P* < 0.01, Fig. 8a–d). In this model, DFO also regulated LPS-induced changes in hepcidin, DMT1, Fpn1, and ferritin (*P* < 0.01, Fig. 8e–i).

## Discussion

The present study investigated the relationship between iron deposition and cognitive impairment after abdominal surgery in a mouse model of POCD. Our data suggest a novel role for iron accumulation in response to surgical trauma in causing neuroinflammation and cognitive dysfunction. Treatment with DFO, an iron chelator, prevented POCD by ameliorating reactive microgliosis and regulating iron homeostasis after surgery.

Neuroinflammation has been described as a hallmark of POCD [29], yet the mechanisms of surgery-induced neuroinflammation remain poorly understood. Dysregulated iron homeostasis is a common feature of many conditions, including disorders of the CNS [30]. In addition, iron and neuroinflammation have been related in pathologies like AD and common neurodegenerative diseases [17]. Iron is essential for several biological activities but requires controlled regulation due to its toxicity when present in abundance [31]. In this study, we found iron concentration significantly elevated in the hippocampus after surgery and long-lasting changes in iron transporting signaling. These findings are consistent with An et al. that suggested iron accumulation and oxidative stress contribute to POCD after splenectomy in rats [32]. Since inflammation is one of the key mechanisms implicated in POCD, we used a model of abdominal surgical model without removal of the spleen to obviate any confounding effects mediated by this immunological organ.

Inflammation and iron homeostasis are tightly regulated. DMT1 has been proposed as a key interface between iron signaling and immunity and is upregulated by pro-inflammatory cytokines like IL-1β [33]. Hepcidin was also found increased in the CNS after surgery, which is consistent with the role of inflammation in triggering iron overload [34]. During chronic inflammation, higher levels of hepcidin and IL-6 have been related to anemia of inflammation, a condition that prevents release of iron from intracellular store [35]. To confirm the role of iron signaling and inflammation in POCD, we used DFO and reported therapeutic effects on both iron transporter signaling and inflammation after surgery, including cytokines like IL-1β and IL-6. Inflammation and iron overload are correlated in chronic inflammatory conditions [36], and systemic cytokines are increased after surgery both in preclinical and human POCD [37–39]. The relationship between systemic changes in iron homeostasis and the CNS is complex and requires further elucidation. Surgery was shown to reduce systemic levels of iron, yet inducing profound expression in the hippocampus [32]. It is possible that these changes observed in the CNS are mediated by pro-inflammatory cytokines affecting iron-related genes at the choroid plexus, the interface between blood and cerebrospinal fluid, by tissue-specific endothelial cells [40]. However, opening of the blood-brain barrier and endothelial dysfunction after surgery [8, 41, 42] makes it possible for the systemic milieu to affect the brain after peripheral trauma, possibly activating directly microglial cells. Notably, DFO administration to control animals had no significant effect on either iron homeostasis or hematological parameters [43].

In the CNS, microglia are critical for surveiling and maintaining homeostasis [44]. Our data support a critical role of microglia activation in POCD both in vivo and in vitro. Surgery activated microglia as noted by CD68 and Iba-1 immunoreactivity up to 14 days after injury, but reactive microgliosis was effectively decreased following DFO treatment. In addition, DFO reduced oxidative stress and cytokine production in microglia cultures. DFO treatment on LPS-stimulated microglia prevented the increase of TNF-α, IL-1β, and IL6, and these cytokines have been shown to increase iron uptake in human monocytes from patients with rheumatoid arthritis [45]. Mechanistically, DFO downregulated levels of gp91phox, the nicotinamide adenine dinucleotide phosphate-oxidase (NADPH) oxidase subunits which are participated in oxidative stress signaling. Dysfunctional iron transporters and excessive free iron induce ROS production affecting redox-sensitive cell signaling and transcription factors [46, 47]. Oxidative stress, including dysregulated NADPH and NADPH oxidase isoform 2 (NOX2) activity, have been recently implicated in POCD [22, 48]. In addition, DFO also inhibited activation of p38 MAPK, which is consistent with the overall reduction in pro-inflammatory cytokines.

Activation of microglia in the hippocampus has been linked to cognitive dysfunction and is causally related to impairment in long-term potentiation [49]. Our data found hippocampal-dependent contextual memory impairment after abdominal exploratory surgery and worse performance in the MWM. This is in-line with previous evidence of hippocampal-dependent cognitive decline in POCD [50]. DFO improved memory impairment by ameliorating inflammation and microglia activation. DFO was previously tested in AD patients to slow progression of dementia [51]. Also, DFO improved cognitive decline, hippocampal inflammation, and cell death after endotoxemia [52]. In our study, we found an effect of DFO on BDNF, which has been implicated in synaptic plasticity and memory processing [53–56]. As we found changes in the calculated area under the curve during MWM training, the effects of DFO on neuronal activity, synaptic plasticity, and BDNF may be critical in alleviating memory deficits. In addition, other mechanisms may contribute to this protective effect, including modulation of neurogenesis, pain, and neurotoxic peptides like β-amyloid [57–59]. Although iron is heterogeneous and widely distributed in every organ including the brain, it remains important to understand why the hippocampus in particular is more susceptible to inflammatory damage.

## Conclusions

In summary, our results indicate that surgery-induced cognitive dysfunction is associated with iron deposition and neuroinflammation. Treatment with an iron chelator, DFO, prevented memory dysfunction in this model by restoring iron homeostasis, neuroinflammation, and oxidative stress. Regulation of microglia activity, including p38 MAPK signaling, and pro-inflammatory cytokines are critical targets to prevent POCD.

## Additional files

Additional file 1: Figure S1. Hippocampal iron content is correlated with neuroinflammation. There is significant correlation between hippocampal iron content and Iba1-indicated microglial activation (*r* = 0.6811, *P* = 0.0147, A), as well as between iron content and CD68 level (*r* = 0.9561, *P* < 0.001, B) on postoperative day 3. (PDF 109 kb)
Additional file 2: Figure S2. Effects of DFO on learning during MWM training. (A) The latency of MWM training trails before surgery. (B) DFO-treated group had significantly reduced the area under the curve during MWM training trials. **P* < 0.05, ***P* < 0.01. Data are expressed as mean ± SD. DFO = deferoxamine; NS = normal saline; AUC = area under the curve. (PDF 36 kb)

## Abbreviations

AD: Alzheimer’s disease
BDNF: Brain-derived neurotropic factor
CNS: Central nervous system
DFO: Deferoxamine
DMT1: Divalent metal transporter-1
ELISA: Enzyme-linked immunosorbent assay
FC: Fear conditioning
Fpn-1: Ferroportin
IL: Interleukin
i.p.: Intraperitoneal
LIP: Labile iron pool
LPS: Lipopolysaccharide
MAPK: Mitogen-activated protein kinase
MDA: Malondialdehyde
MWM: Morris water maze
NADPH: Nicotinamide adenine dinucleotide phosphate-oxidase
NF-kB: Nuclear factor-kappa B
NOX2: NADPH oxidase isoform 2
OCT: Optimal cutting temperature compound
PFA: Paraformaldehyde
POCD: Postoperative cognitive dysfunction
ROS: Reactive oxygen species
TNF-α: Tumor necrosis factor-alpha
