# Supplementary figures and images for: Deferoxamine regulates neuroinflammation and iron homeostasis in a mouse model of postoperative cognitive dysfunction
Source: J Neuroinflammation. 2016 Oct 12;13:268. doi: 10.1186/s12974-016-0740-2 (PMC5062909; doi:10.1186/s12974-016-0740-2)

# Supplemental Figure 1

A

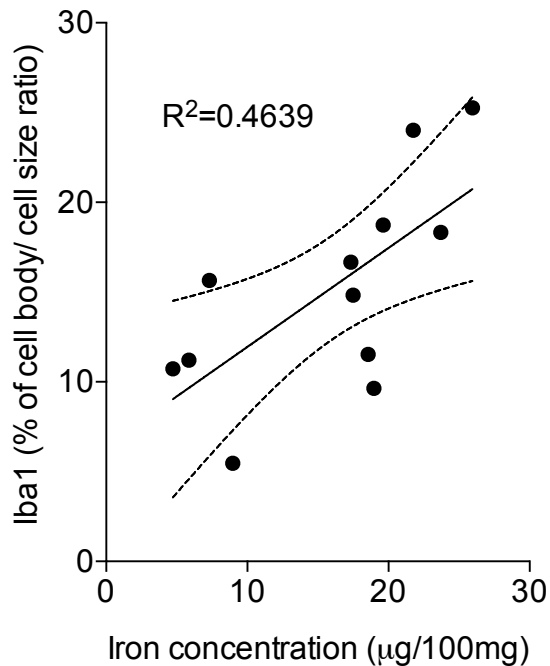

B

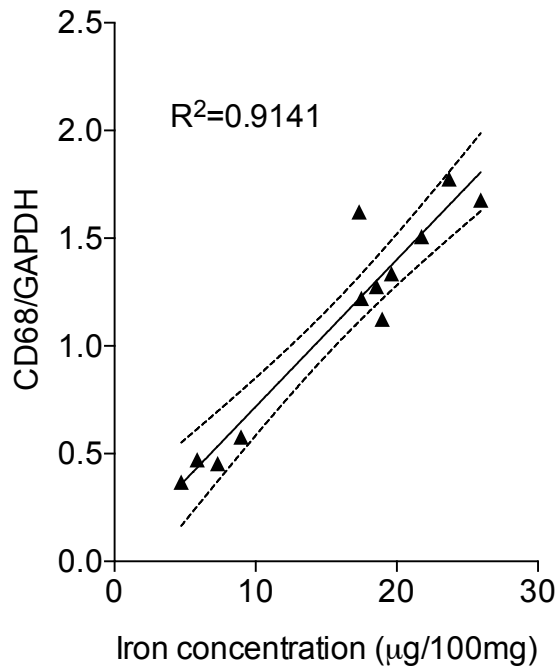

Supplement: Additional file 1: Figure S1. — Hippocampal iron content is correlated with neuroinflammation. There is significant correlation between hippocampal iron content and Iba1-indicated microglial activation (r = 0.6811, P = 0.0147, A), as well as between iron content and CD68 level (r = 0.9561, P < 0.001, B) on postoperative day 3. (PDF 109 kb) [file 12974_2016_740_MOESM1_ESM.pdf]

# Supplementa Figure 2.

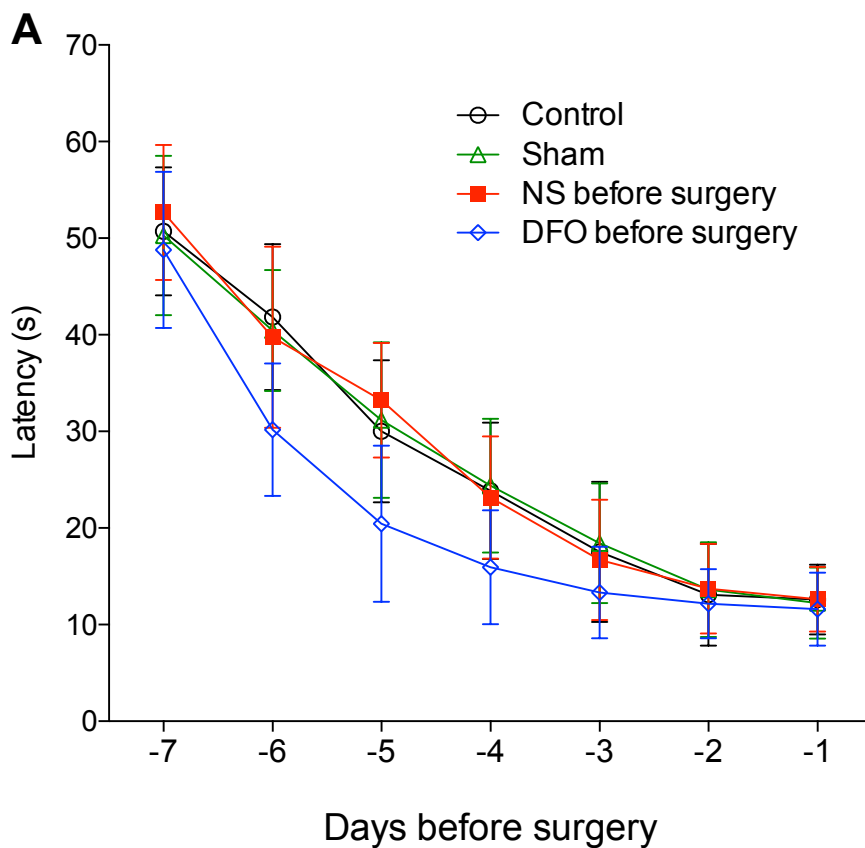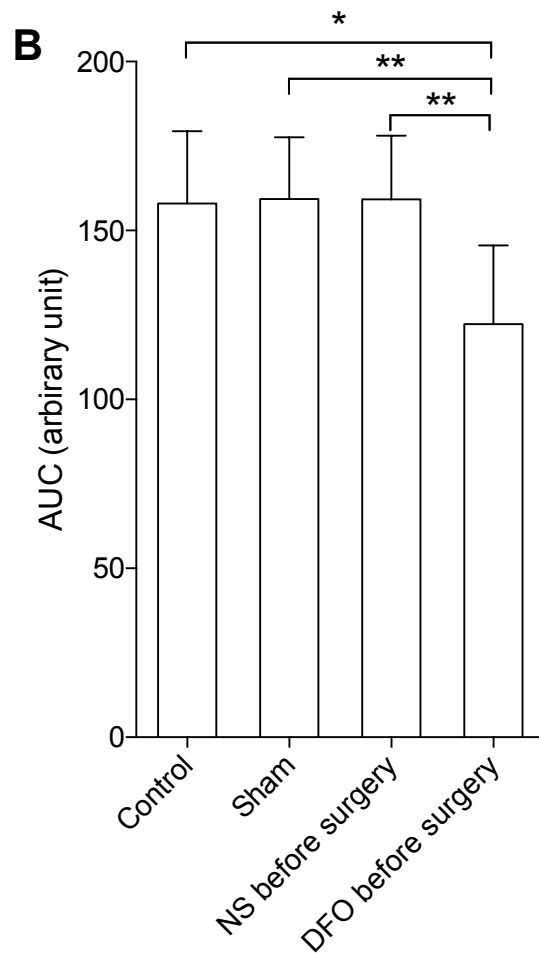

Supplement: Additional file 2: Figure S2. — Effects of DFO on learning during MWM training. (A) The latency of MWM training trails before surgery. (B) DFO-treated group had significantly reduced the area under the curve during MWM training trials. *P < 0.05, **P < 0.01. Data are expressed as mean ± SD. DFO = deferoxamine; NS = normal saline; AUC = area under the curve. (PDF 36 kb) [file 12974_2016_740_MOESM2_ESM.pdf]
